# In Vivo Analysis of Protein–Protein Interactions with Bioluminescence Resonance Energy Transfer (BRET): Progress and Prospects

**DOI:** 10.3390/ijms17101704

**Published:** 2016-10-11

**Authors:** Sihuai Sun, Xiaobing Yang, Yao Wang, Xihui Shen

**Affiliations:** State Key Laboratory of Crop Stress Biology for Arid Areas and College of Life Sciences, Northwest A&F University, Yangling 712100, Shaanxi, China; sihuai.sun14@alumni.imperial.ac.uk (S.S.); bing3698@163.com (X.Y.); wangyao@nwsuaf.edu.cn (Y.W.)

**Keywords:** protein–protein interactions, bioluminescence, fluorescence, Bioluminescence Resonance Energy Transfer, Förster Resonance Energy Transfer, NanoLuc, bacterial luciferase, protein dynamics

## Abstract

Proteins are the elementary machinery of life, and their functions are carried out mostly by molecular interactions. Among those interactions, protein–protein interactions (PPIs) are the most important as they participate in or mediate all essential biological processes. However, many common methods for PPI investigations are slightly unreliable and suffer from various limitations, especially in the studies of dynamic PPIs. To solve this problem, a method called Bioluminescence Resonance Energy Transfer (BRET) was developed about seventeen years ago. Since then, BRET has evolved into a whole class of methods that can be used to survey virtually any kinds of PPIs. Compared to many traditional methods, BRET is highly sensitive, reliable, easy to perform, and relatively inexpensive. However, most importantly, it can be done in vivo and allows the real-time monitoring of dynamic PPIs with the easily detectable light signal, which is extremely valuable for the PPI functional research. This review will take a comprehensive look at this powerful technique, including its principles, comparisons with other methods, experimental approaches, classifications, applications, early developments, recent progress, and prospects.

## 1. Introduction

In biological systems, it is fair to say that almost all molecules achieve their functions by interacting with each other. As the fundamental machinery of life, proteins without doubt are the most important kinds of molecules in cells and involved in interactions that account for many essential biological processes such as catalysing reactions and signal transductions. Although there are many types of interactions that proteins can participate in, for example, protein–DNA interactions and protein–substrate interactions, in most of the scenarios, studies on these interactions often lead to the most common and important type of interaction—Protein–Protein Interactions (PPIs).

Protein–protein interactions are often considered the backbone of the “interactome”, which is the whole set of molecular interactions that exist in a cell [1]. In an interactome, molecular interactions connect all kinds of molecules to form a giant and sophisticated network, representing all the biological processes in that cell, and it is crucial for the understanding of life at the fundamental level. As most part of the interactome is comprised of interactions among proteins, PPIs mediate many other types of interactions to bring them into a single interaction network. For instance, many transcription factors (in protein–DNA interactions) carry out their functions in joint with other proteins in a complex [2], and many receptors (in protein–ligand interactions) using PPIs in the downstream signalling cascades [3]. In theory, all PPIs and their properties can be precisely predicted from genomic information via computational approaches, however the practical accuracy and applicable scope are limited due to computational power and methodology. Therefore, reliable and detailed PPI data can only be obtained or verified by experiments, and having proper methods to do so is extremely important in any biological researches that involve molecular scale processes.

There are many techniques available for the investigation of PPIs, each has its own strengths and weaknesses, but only a handful of them are commonly used owing to their overall performance and usability. According to statistics from MINT, an online database that has collected more than 240,000 experimentally verified PPIs through literature mining, about 46% of the evidences of interactions were detected by Yeast Two-Hybrid (Y2H) method, 21% by Co-Immunoprecipitation (Co-IP), and 25% by Tandem Affinity Purification (TAP) [4]. Y2H is the most commonly used method, and since its first development, it has been modified into many variants, became a versatile method for detecting PPIs of all kinds. It utilizes the fact that many eukaryotic transcription factors have two distinct domains, both of which are required for the activation of transcription, so if these two domains separately attach onto two proteins of interest, the system will able to indicate whether the two proteins interact through the activation of a related reporter gene [5,6]. Y2H can be easily achieved with basic molecular techniques, suitable for even weak PPIs, and is sensitive. However, it can only be done in vivo, has zero or negligible responses to dynamic interactions, and requires test proteins to be nucleus localizable. Co-IP and TAP are also popular methods; both of them rely on stable bindings between proteins and developed from protein purification methods. They use antibodies to bind to a protein of interest, while the antibodies are fixed onto something that can be easily manipulated such as beads or matrixes, so by separating the beads or matrixes from solutions of proteins, the protein of interest will be pulled out, as well as other proteins that it has stable interactions with, which will then be identified by techniques such as mass spectrometry [6]. These two methods have the advantages of detecting multiple PPIs at one time, and intrinsically high-throughput, which is helpful in applications such as drug development for finding potential binding partners of specific proteins. Nevertheless, their scope of application is even more confined than Y2H, as they can only be done in vitro and are not suitable for weak or transient PPIs and more detailed PPI studies.

Due to the limitations of these dominating methods, new and improved methods are constantly developed to fulfil the needs of increasingly important PPI research. Many of them are quite good for their applications, for example, Protein Microarray is brilliant for tracking and functional determination of PPIs on a large scale [7], and NMR (Nuclear Magnetic Resonance) spectroscopy can be used to study PPIs in extremely fine detail [8]. However, many of those methods need specialized or expensive equipment to be performed, and sometimes require specific knowledge to analyse the result [6,9]. Therefore, classic but simple methods like Y2H are still being widely used in many laboratories routinely for PPI detection. This paper will review a method called Bioluminescence Resonance Energy Transfer (BRET), which has been around for quite a few years, but with recent advances, it shows the superior potential of being a powerful yet simple method and an excellent alternative of Y2H for general binary PPI studies. Akin to Y2H, the procedure of BRET is very straightforward and the result is easy to interpret, and it can be done with basic molecular techniques at relatively low costs, thus very accessible for many laboratories. Furthermore, unlike Y2H, BRET does not require cell activities to show the signal, so it is achievable both in vivo and in vitro, and can be used on proteins with no nucleus localization. Most importantly, the greatest distinctive feature of BRET assay is that it has a quick and reversible response to dynamic PPIs, and employs the easily measurable light as readout signal, which is extremely valuable for real-time monitoring of PPIs for functional and regulatory research or other utility applications. In addition, with appropriate equipment, it can even be used as an imaging technique to map and study PPI localization.

## 2. Principles of Bioluminescence Resonance Energy Transfer (BRET) and Comparison with Related Methods

### 2.1. Principles of BRET

All intermolecular forces work at short distance, and at perspective of a single molecule in a cell, the molecule can only experience forces from its neighbouring molecules, causing it to randomly move around and make numerous collisions in its path. In most cases, these collisions will simply end up with molecules quickly bouncing off each other, and cause no biological effect. However, in the case of protein–protein interaction, two proteins collide by the correct angle and momentum, and stick to one another for a either short or long period of time after collision to allow further events to happen. Although the actual movements of proteins are still random and caused by temperature, statistically speaking, the existence of PPIs will bring proteins to closer distance, which is what BRET and many other methods are trying to detect.

**Figure 1:**
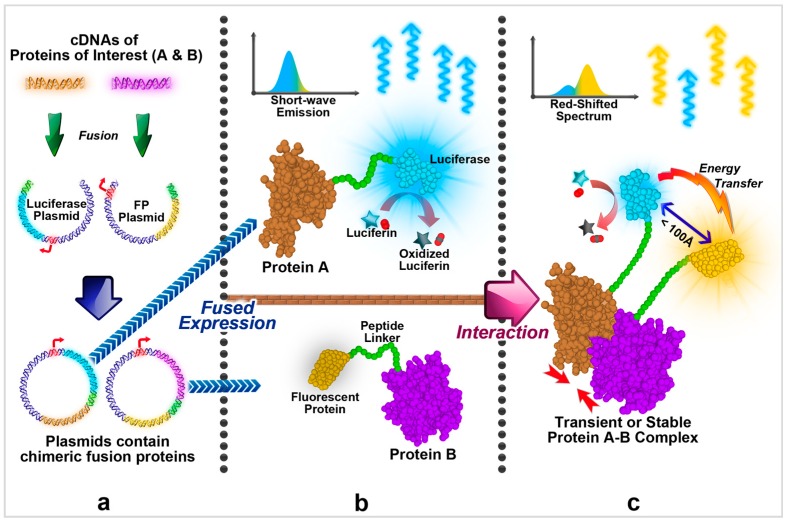
Principles of BRET assay. Arrows show the oversimplified procedure of using BRET to detect the interaction between two proteins: (**a**) The cDNAs of proteins of interest (Protein A and B) are separately fused with genes of luciferase and fluorescent protein, and co-expressed as tagged chimeric proteins; (**b**) luciferase oxidizes luciferin, giving off high-frequency light, while the fluorescent protein remains inactive; (**c**) interactions between Protein A and Protein B bring luciferase and fluorescent protein to close proximity, then fluorescent protein draws energy from luciferase and generates lower frequency light, causing the easily observable frequency shift in spectrum.

In BRET, two proteins of interest, which are called “bait” and “prey”, are separately attached to a luciferase and a fluorescent protein (FP). Both the luciferase and fluorescent protein are proteins that can generate light, however, luciferase generates light by oxidizing luciferin [10], while FP requires external excitations such as higher frequency light to glow. Therefore, in the presence of luciferin but no external excitation, normally only the luciferase can constantly produce light, which is the case when there is no direct interaction between the proteins of interest. On the other hand, if the two proteins of interest can interact, the interaction will bring them to close proximity, as well as their linked luciferase and FP, and a phenomenon called Förster Resonance Energy Transfer (abbreviated to FRET or RET) can occur [11]. During the RET event, excited electron energy on luciferase (donor) is transferred to FP (acceptor), causing the excitation of FP and its production of light. As the frequency of produced light is different between luciferase and FP, the energy transfer event can be easily observed and quantified, indicating the presence of interaction between the proteins of interest (Figure 1).

Many types of luciferases and fluorescent proteins exist, each having its own physical properties and optical characteristics, and BRET can be done using various combinations. Depending on the choice, each combination may have certain advantages over the others; for example, pairs that contain long wavelength emitting FPs perform better in deep layer PPI detections. These different selections lead to BRET systems with different advantages, and when studies claim to have invented new BRET methods, it usually means they have found new luciferase–FP pairs that have certain advantages, rather than any changes to BRET principles or procedures.

The energy transfer between luciferase and fluorescent protein can occur effectively only when they are in the 100 Å (10 nm) range, which fits most biological interactions including PPIs well [12]. This range is also much smaller than the wavelength of emitted photons, so the energy transfer is through a radiationless dipole–dipole coupling mechanism at the quantum level rather than the reabsorption of photons [13]. This make the method very sensitive to distance changes, and, according to Scholes [14], the RET efficiency (ratio of transferred excitations to total donor excitations) is inversely related to 6th-power of separation distance, which is so sensitive that it even allows the detection of protein conformational changes with few modifications to PPI BRET. However, even though there is no real photon involved in the transfer process, an effective RET system requires donor emission spectrum overlaps with acceptor excitation spectrum, and, combined with few other factors, they determine the Förster distance (where RET efficiency is 50%) of a RET system, which will greatly impact the signal quality [14]. Therefore, RET is a conditional and directional process, and validation is required for different luciferase–FP combinations used in different BRET variants.

### 2.2. FRET and Protein-Fragment Complementation Assays

RET is a general phenomenon that can occur not only between luciferase and fluorescent protein, but also between two different fluorescent proteins, or among many other light-sensitive molecules or even inorganic compounds such as quantum dots [15]. Therefore, the same energy transfer principle can be adopted in many other techniques for various applications. For PPI studies, there is an alternative sister method of BRET that differs mainly from energy donor part, where the luciferase donor in BRET is replaced with another fluorescent protein and the whole system is usually referred to as Fluorescence Resonance Energy Transfer. This system is also abbreviated to “FRET”, which is the same abbreviation for Förster Resonance Energy Transfer, so in many studies, “FRET” may imply either the technique or the mechanism. In this review, “FRET” is used to describe the technique, and “RET” is used for the corresponding mechanism. FRET was actually developed earlier than BRET, and, in FRET, the fluorescent protein donor is activated by externally applied excitation light, so, during observation, a background light, which may negatively affect the signal, is inevitable [16], which led to the development of BRET.

Both FRET and BRET are very similar and comparable to another category of methods called Protein-fragment Complementation Assays (PCAs), which represent the general idea of using halved functional protein to tag the proteins of interest and observe functional recovery of halved protein to detect the presence of interactions [17]. Many popular methods for studying PPIs nowadays belong to this category, including Y2H. Another representative method of PCAs is Bimolecular Fluorescence Complementation (BiFC), which uses halved fluorescent protein to tag proteins of interests, and when proteins of interest can interact, the halved and deactivated fluorescent protein parts will be brought together and the fluorescence can be restored [18]. Similarly, the principle can be applied to halved luciferases [19,20], proteases [21], kinases [22], ubiquitin [23], etc. As all these PCAs methods adopt the same underlying concept, they share some common characteristics such as they need the construction of transgenic vectors for fusion proteins, but each method may have its own strengths to fit in different situations.

### 2.3. Compare BRET with FRET and Mainstream Protein-Fragment Complementation Assays (PCAs)

Though BRET and FRET are generally not classified as PCAs methods, these RET-based approaches and PCAs are comparable, and both BRET and FRET have the full potentials that ideal PCAs can reach without the significant drawbacks many PCAs suffer. For example, typical Y2H method requires cell activities and is limited to nucleus localizable proteins; binding of FP fragments in BiFC is not reversible [18]; and DHFR (Dihydrofolate reductase) PCA cannot ensure negative result [24]. In contrast, the RET mechanism is totally physical, and light signal is also physical, therefore, it does not require any cell activity for signal readout, which allows in vitro assay, and, more importantly, it also allows the RET signal to be instantly generated without transcription or other forms of delays when proteins of interest interact. Furthermore, as there is no intrinsic relation between luciferase and FP, the chance of inter-tag PPI is very low in BRET and FRET compared to PCAs, so this minimizes the false positives and makes the mechanism fully reversible. This reversibility combined with the instantaneous signal response makes the BRET and FRET incredibly useful in investigating the dynamics of PPIs, which is essential for the understanding of PPI functions.

As more and more PPIs have been identified each year by various methods, their functional studies are becoming increasingly important for many research and applications, and both BRET and FRET prevail with their versatility and overall performance over PCAs (Table 1). However, as BRET employs the endogenous light source, it has some distinct advantages compared to FRET. The main advantage is that BRET has almost no background luminescence or false signals that are generated by all kinds of unintentional excitations by external light, which greatly increases the signal-to-noise ratio, hence increases the sensitivity and accuracy. Moreover, the absence of external light in BRET also avoids problems such as photobleaching of light sensitive molecules, photo-toxicity [25], and inhomogeneous excitation due to light scattering [16], thus leading to more stable and reliable signal in many situations. Additionally, without the need for external excitation, the equipment required in BRET can be simpler and cheaper, and BRET is more suitable and adaptable for high throughput screening than FRET [25].

## 3. General Approach of Conducting BRET Assays

Many BRET systems have been reported for studying PPIs, but they usually differ only in the choice of luciferase–FP pairs or sometimes substrates, and the main procedures are essentially the same. This section will briefly illustrate the common procedures for performing the standard BRET assays in order to provide a basis for subsequent discussions. Readers who want to perform an actual assay should find more detailed protocols elsewhere in accordance with their aims and situations.

### 3.1. Vector Design and Acquiring

The first step of BRET assay is to obtain luciferase or FP-tagged proteins. However, as it can be problematic to tag the matured proteins, this is almost exclusively done by gene fusion to directly produce chimeric proteins that contain the proteins of interest and corresponding tags in living cells. In the process of gene fusion, suitable expression vectors are constructed, where corresponding cDNA sequences are joined together with a short linker in between, and the stop codon before the linker is removed to allow merged expression [26]. The linker in the vector is designed to be expressed as a short but flexible polypeptide, so that separated protein parts can fold independently without too much impact. According to Chen et al., the optimal linker for the purpose should be around 10–15 aa length, and consist of mainly glycine and serine residues, such as (GGGGS)_3_, which is the most commonly used [27]. Another aspect that needs to be considered in vector design is fusion topology, it is the order or arrangement of each protein part in the fusion, and many research have shown that this can greatly alter the activity of fused proteins in certain circumstances [11,28]. There can be up to eight different fusion patterns for a set of luciferase–FP tags with two proteins of interest, and the vector should be designed to minimize the steric constraints for its protein parts. However, currently there is no reliable way to predict which one would be the best configuration, so empirical validations should be performed on each protein with both N and C terminus fusions when possible [17]. Depending on the situation, controls may be required in some assays; for these, positive control is usually a direct fusion of luciferase donor and FP acceptor, and negative control can be set by test interactions between one fusion protein and a counterpart unfused luciferase or FP tag.

BRET can be conducted in almost any biological system, so the actual base vector and promoter choices really depend on the host cells, which further depend on the proteins that need to be tested. The selected host cells should have the ability to do all the necessary post-translational modifications for the proteins of interest and tags, and fusion proteins′ expression levels should close to the original proteins′ natural levels, so it is advisable to use native cells and endogenous promoters for BRET assays. However, in some BRET systems, the luciferases are not bright enough to generate detectable signals at physiological concentration, so some overexpression may be needed, but it should be noted that too much overexpression must be avoided as it can lead to non-specific BRET and cause false positive [29]. Recombinant vectors can be obtained by traditional molecular methods, but, due to the multiple modifications, the actual procedures can be complicated and error prone, so the constructed vectors often need to be sequenced to ensure accuracy in many researches [28,30,31]. Alternatively, with the development of synthetic biology, many companies are now offering custom gene synthesis up to 10,000 bp with guaranteed accuracy and speed, as the price is decreasing every year, this can be a good option for many future studies.

### 3.2. Fusion Expression and Signal Observation

Once the recombinant vectors have been obtained, they need to be transferred into host cells for expression. The methods for this can be vary and are depending on cell types, but as each vector construction usually codes for only one fusion protein, two different vectors need to be inserted into the same cells, so selection methods, as well as vector compatibility should be considered in advance during experimental design. However, this can be less important when adopting transient transfection approach, especially for some high-throughput adaptations. The approach requires multiple different parallel transfections to be set up in an observation, and in each setup, two kinds of expression vectors are mixed together with a defined concentration and used to transfect cell cultures prepared from the same cell batch [32]. These parallel setups must include vector pairs intend for BRET signal as well as positive and negative controls and other fusion topologies, and the actual result should be concluded by comparing signals from these different transfections. This approach will generate transient transfections that will be lost over time, so necessary expression inductions and observation should be performed 24–72 h after transfection [26].

Fusion proteins expressed by cell cultures can be extracted and purified for in vitro assays, but that will involve additional works, so BRET signal is mostly observed in vivo for both convenience and the iconic features it brings. To start an observation, substrate must be supplied to luciferase. For some BRET systems, it is possible to engineer the host cell to produce substrate endogenously, but in most scenarios, the substrate should be added externally. The substrate is usually membrane permeable but at a low rate [33], hence minutes to hours incubation time is required before signal readout if there is no endogenous substrate. BRET signals can be observed using fluorescence microscope, microplate luminometer, single-tube luminometer, or scanning spectrometer [26]. Scanning spectrometer can give detailed spectrum information, which is very helpful if emissions from luciferase and FP are wide or close by, and it is also valuable for data credibility as many potential errors will be detected if they exist; however, the scanning process can be slow and the equipment may not suitable for some culture types. Fluorescence microscope and luminometer are more general and straightforward; by using different filters, emissions from luciferase and FP can be distinguished and recorded, but ideal filters may not always available for each BRET systems and it can result in reduced data quality.

### 3.3. Signal Analysis

The presence of PPI is indicated by BRET ratio (BRET signal), which is defined as the light intensity of FP emission relative to luciferase emission [34]. This ratio should not be confused with RET efficiency that can only range from 0 to 1; the BRET ratio can be any value above 0. The light intensity from luciferase is directly measured using filters or calculated by integral, but FP emission must be corrected in the calculation of BRET ratio as there can be an overlap of luciferase emission at FP′s central wavelength. Mathematically, the actual BRET ratio can be calculated by subtracting the directly measured BRET ratio with a background BRET ratio, where the background ratio can be measured using cells that only express the corresponding luciferase [34]. This calculated BRET ratio can reflect the relative interaction strength between tags labelled proteins of interest, and in general, the ratio should be at least 0.1 to be considered significant as the evidence of interaction.

In PPI dynamics studies, the BRET ratio can act as an ideal real-time indicator for the relative interaction strength between proteins when their expression level are fixed, so by monitoring its changes while altering external conditions, this can be used to investigate PPI regulations and functions [28]. However, BRET ratio of the same PPI can be variable depending on construct and expression level, so it cannot be used as a measure of absolute interaction strength and the ratios from different experiments are usually not comparable. Although some reports have been made on quantitative BRET (qBRET) assays, which involves plotting the BRET signal of certain PPI at different acceptor/donor expression ratios and then using derived BRET_50_ value (acceptor/donor ratio where BRET signal reaches half maximum) to indicate the protein binding affinity of this particular PPI (lower BRET_50_ means stronger tendency of interact) [32,34], such methods are flawed, as proportioned BRET signals are still not able to reflect the actual binding state of proteins in a constant way, especially when RET efficiency is relatively high. Therefore, to accurately compare different sets of PPIs, the absolute protein interaction strength, which is often quantified by dissociation constant *K*_d_, should be measured by some biophysical methods such as Isothermal Titration Calorimetry (ITC).

### 3.4. BRET Imaging

Light is one the most generic forms of signal and it can be captured by numerous kinds of devices; by using digital cameras in BRET detection, the localization of PPIs can be studied, and it can also help in many other applications. However, in many BRET systems, luciferase has a very low quantum yield and generates very dim lights; it is so dim that the photons emitted are below the detection threshold of many common CMOS (complementary metal-oxide-semiconductor) or CCD (charge-coupled device) sensors, so BRET imaging is traditionally unpopular [29]. Solving the problem requires either brighter BRET systems or better photon detection techniques. A number of BRET systems that are significantly brighter have been developed in recent years, and many of them are based on NanoLuc^®^ luciferase, which is an artificially engineered luciferase that is about 100 times brighter than many traditionally used luciferases [35]. For these systems, it is possible to use conventional cameras to do the imaging, but usually limited at tissue or culture level. These brighter systems can partially solve the problem but it is not universal, so in most of cases, BRET imaging is achieved with the more general approach of using very sensitive cameras. CCD sensor can be made more sensitive with simple cooling apparatus such as Peltier cooler, so it was popular in early years for the task [36]. However, when it comes to single cell or subcellular level, the amount of photons that can be captured is much less, and cooled CCD sensor will struggle in this situation. Electron Bombardment CCD (EB-CCD) can reach much better sensitivity with an amplification field installed in front of a normal CCD sensor, and it has shown its power in many recent researches for cell level BRET imaging [37,38].

No matter what system or camera is used, the imaging process should be performed in a light-tight box to avoid any external interference. Cameras can be directly attached to appropriate microscope for observation, but, as there are two emission wavelengths from the sample, a camera coupled with a dual-view image splitter and filters to record signals at each wavelength simultaneously is often used [37]. The exposure time is dependent on sample type, camera, and BRET system: when using EB-CCD camera, it can be less than a second for most types of samples, but this may stretch to a few minutes if deep layer imaging is involved. To reduce the exposure time and enable real-time and high spatial resolution monitoring of PPIs, both high brightness BRET system and high sensitivity camera should be used [38].

## 4. Developed BRET Systems and Their Applications

### 4.1. Overview

Even though BRET assay is a generic and flexible method that can be achieved with many different kinds of donors and acceptors, researchers tend to use previously reported and well-validated BRET systems for regular PPI studies. Since the earliest BRET assay reported by Xu et al. in 1999 [39], several, but less than a dozen, BRET systems have been developed and commonly used. Many of them are improvements made from previous systems by altering the substrate, FP acceptor, and, less commonly, luciferase donor. When these systems are first published, authors usually give them each a name for discrimination; however, as no standard nomenclature has ever been established, these names do not always follow a rigid rule or pattern [34], and, even worse, sometimes different authors may give the same name for different systems or same system different names. Therefore, this should be taken into account when interpreting different literature. A summary of these systems is compiled in Table 2 for reference.

### 4.2. Renilla Luciferase Based BRET Systems

Luciferase is the core part of a BRET system; among all those developed systems, Renilla luciferase (Rluc) and its variants have been the most prevalent since the earliest BRET^1^ was published. The natural form of Rluc originates from *Renilla reniformis*, a small marine animal species living in shallow sea areas that will glow green when disturbed. Rluc is a medium sized luciferase at around 36 kDa, and, in the presence of oxygen, it will oxidize its substrate coelenterazine into coelenteramide and generate blue light that peaks at 480 nm [51]. A special feature of Rluc is that its emission can be altered by only changing the substrate, and, by using different kinds of artificially modified coelenterazines, Rluc based BRET systems can be modified to adapt different situations (Table 2).

BRET^1^ is the initial form of Rluc based BRET systems; it uses wild type Rluc as donor, eYFP as acceptor, and native coelenterazine as substrate [39]. This system is generally good with decent brightness and high substrate stability; however, as Rluc emission can cover a relatively wide spectrum, there is a significant emission overlap between Rluc and eYFP, which reduces the signal to noise ratio. To increase the signal quality, BRET^2^ was developed with coelenterazine 400a (also called DeepBlueC) as substrate to blue shift the Rluc′s peak emission to 395 nm [40], and, accordingly, GPF^2^ is used as acceptor, because the eYFP will not be excited too much with the blue shifted Rluc emission. BRET^2^ can achieve a peak emission separation of 115 nm, which is excellent compared to other systems, but, on the other hand, DeepBlueC has a very low quantum yield and unstable, which makes BRET^2^ very dim and lights will quickly die away in just few seconds [41]. This drawback led to the development of enhanced BRET^2^ (eBRET^2^), which uses Rluc8, a Rluc mutant, to replace native Rluc in BRET^2^. Compared to native Rluc, Rluc8 is significantly brighter and more stable while it keeps the other essential properties of Rluc [52]; therefore, Rluc8 can be used not only in BRET^2^, but also in BRET^1^ and other subsequently developed systems.

BRET^1^ and eBRET^2^ are the most popular BRET systems being used in many PPI studies, with their significant advantages in investigating dynamic PPIs, many of these research are about G protein-coupled receptors (GPCRs). GPCRs are the most important class of receptors in eukaryotes; they are monomeric transmembrane proteins, very diverse, carrying a large variety of essential functions, and the target of more than 50% of drugs on the market [53]. As the GPCRs′ role is achieved by a series of dynamic interacting processes among external ligands and internal pathways, BRET is one of the essential tools for GPCRs studies, and helped in the accomplishments of many important researches [40,53,54,55,56,57,58]. Nevertheless, when it comes to the BRET imaging and deep layer PPI detection, the use of BRET^1^ and eBRET^2^ is greatly hindered by the systems′ short wave emissions as most tissues have a significant absorbance at this frequency range.

To make BRET more efficient for BRET imaging, it requires BRET systems to operate at longer wavelengths; thus, BRET^3^ was developed by using mOrange as acceptor FP, which has a peak emission at 564 nm [42]. However, this only partially solves the problem, as Rluc8 is still emits at the blue frequency, so red shifting of Rluc emission has become the next improvement. This was done using another coelenterazine analogue called coelenterazine-v, which shifts the Rluc8 emission to 515 nm (BRET3.1), and then the more red-shifted TagRFP can be used as acceptor (BRET4.1) [46]. After the discovery of Rluc8 mutation in 2007, a red-shifted Rluc8.6 variant was also published [52], so Rluc8.6 was used as well to solve the problem, and, combined with different FPs and substrates, BRET5–BRET8 have been created [46,47]. These red-shifted BRET systems are widely tested and proven to be efficient with varieties of organisms including mice, plants, and human tissue cells.

As Rluc is one of the most exploited luciferases, there are still plenty of different synthetic substrates available besides those mentioned above [59], and many of these substrates can be used in BRET to gain some other benefits. For example, coelenterazine-h is much brighter than the native one, and, as it does not change Rluc′s spectral properties, many BRET systems that were using native coelenterazine are now actually performed with coelenterazine-h, even though it has a shorter luminous period. Some other examples include EnduRen, which is very stable and can be used to achieve much longer observation time (extended BRET, eBRET) [44]; ViviRen, which is even brighter than coelenterazine-h and have a lower autoluminescence, but the high brightness only last for a short period [45].

### 4.3. BRET with Firefly and Gaussia Luciferases

Though not very common in BRET systems, Firefly luciferase (Fluc) is actually the most extensively researched and exploited luciferase in biology: it has been used as a reporter gene, bioluminescence tag, ATP sensor, epigenome profiler, and other roles in numerous applications. Unlike Rluc, Fluc oxidizes d-luciferin to generate orange light that peaks at 565 nm and the emission has a similar but longer lasting brightness compared to Rluc. This naturally-occurring long wavelength emission can be useful in deep layer PPIs detection, so a BRET system using Fluc as donor and DsRed as acceptor was developed in the early year [60], and referred to as BRET^3^ in some literature (the same name is also used for a previously mentioned Rluc system). However, this system failed to become popular like many Rluc based ones, and Fluc is still very rare in today′s BRET systems. This is due to several reasons: First, the Fluc has a bulky size of 62 kDa [61], which is almost as twice as big of Rluc; this may have a negative impact to the tagged proteins and also makes it hard to manipulate. Second, the activity of Fluc is ATP dependent and requires magnesium cation as co-factor, which has further limited its application [62]. Lastly, the emission of Fluc is sensitive to temperature and ionic strength, and the spectrum will be red shifted if temperature increases, which means the Förster distance is not constant, and the result will be inaccurate or misleading [43].

Those disadvantages set back the application of Fluc in BRET, and Rluc still dominates the method. Nevertheless, Rluc is not perfect and researchers are continuously questing for better luciferase options, and, as usual, such discoveries come from nature. A marine copepod species called *Gaussia princeps* is known for its unusual feature of leaving a bright luminous trail when they swim quickly [63], and, in 2002, researchers successfully isolated the source responsible for the phenomenon, Gaussia luciferase (Gluc), and its corresponding cDNA [64]. Gluc has a size of 20 kDa, which is one of the smallest luciferases known, and similar to Rluc, it also uses coelenterazine as substrate and generates 475 nm light. However, more importantly, Gluc is very bright, and experiments show that when expressed in mammalian cells under similar conditions, Gluc is 100-fold brighter than Rluc [65]. The small size and brightness make Gluc an attractive luciferase for BRET assay, and Li et al. reported that the codon optimized Gluc (hGluc) can be used in BRET^1^ to increase sensitivity [66]; later they also developed a red-shifted system with tdTomato as FP [48]. However, currently, Gluc works best only with native coelenterazine, and common alternative substrates for Rluc such as coelenterazine-h and ViviRen are not compatible with Gluc [67]. As native coelenterazine is relatively unstable (autoluminescence) and there is no red-shifted Gluc mutant available yet, more improvements are needed to promote Gluc for wider and more versatile use.

### 4.4. NanoBRET

An ideal luciferase should be monomeric, small, bright, stable, versatile, and have a minimum requirement to environment and substrate; however, such perfect luciferase seems hard to be found from nature, though Gluc is a good one. To acquire better luciferase, artificial protein engineering strategies including both rational design and directed evolution are needed. Following the path, an artificially engineered luciferase called NanoLuc^®^ (Nluc) was developed a few years ago by the biotech company Promega [35], and, with its excellent properties, this new luciferase has quickly been adopted by many researchers for BRET assays [49]. Nluc is developed from the naturally-occurring Oplophorus luciferase (Oluc), which is a tetrameric luciferase that is seven times brighter than Rluc but with a huge size of 106 kDa [68,69]. Through sequence and functional analysis, the company extracted the core part of the Oluc, and further engineered it to the miniature 19 kDa size Nluc but with a brightness that is 100 times that of Rluc [70].

Compared to Gluc, Nluc is a little bit smaller and similar in brightness, but it has some other good properties that outperform current Gluc. Furimazine is the substrate used by Nluc (λ_Em_ = 460 nm), which is very stable with a half-life of more than 2 h compared to 25 min for the native coelenterazine used by Gluc. This stability permits not only longer observation, but also much lower autoluminescence [70]. The low autoluminescence can give a higher signal to noise ratio and thus better sensitivity; an experiment to directly compare the luminescence from Nluc and Gluc systems illustrated that the Nluc system has 10 times higher signal to noise ratio, which will enable ultra-sensitive assays [71]. Versatility is another excellent characteristic of Nluc: it is confirmed to work in a wide range of pH and temperature, and does not need any post-translational modifications, so it can be easily incorporated into almost any organisms including human and *E. coli* [70]. It also does not have any targeting sequence or compartment bias, whereas Gluc is naturally secreting targeted and thus needs confirmation or modification for wider use [72].

In actual NanoBRET systems, the high brightness and low background luminescence make NanoLuc^®^ able to be used not only in conjunction with normal FPs, but also HaloTag^®^ for better flexibility [49]. HaloTag is a specially engineered protein that can be granted with different functions by simple ligand reactions [73]. It is not FP but can be made fluorescent by various ligand dyes. In such BRET system, HaloTag is treated as FP to fuse with the protein of interest, then right before the normal signal detection, it is granted with chosen fluorescent ability by incubating cell culture with appropriate labelling reagent [49]. By using HaloTag in BRET, it allows researchers to try and use different acceptor dyes, for example, red emitting dye for better signal separation or deep layer PPI and yellow emitting dye for better RET efficiency, with the same plasmid construct. However, it should be noted that the Nluc-HaloTag BRET system usually has a low-level BRET ratio compared to normal systems, so the result must be interpreted relatively [49]. Overall, Nluc based BRET systems possess excellent sensitivity, stability, veracity, and versatility, make them the superior replacement for many classic Rluc based BRET systems. The only major drawback of Nluc is that it does not have a red-shifted version yet, so some Rluc or Fluc systems are still useful for in vivo imaging tasks.

### 4.5. LuxBRET in Bacteria

Although Rluc and Nluc based BRET systems are very effective and adaptive in most cases, such assays usually involve very high-cost substrates. For example, 100 mg native coelenterazine cost $50,000 [74], 100 mg EnduRen™ (Madison, WI, USA) cost $23,600 [75], 100 mg DeepBlueC™ (Fremont, CA, USA) cost $20,900 [76], and furimazine is proprietary and sold in kits starting from $143 (only enough for one use) [77]. In contrast, d-luciferin used by Fluc is much cheaper at $1,670 for 100 mg [78], which is one of the reasons why Fluc is more popular than Rluc in general luminescence assays, but the price can still be considered as high. Additionally, these substrates are sensitive to light, heat, and oxygen, required fastidious storage conditions, and need constant injection for prolonged observation [79]. Therefore, it would be very valuable if BRET could be done without adding substrates, or with more economical alternatives.

**Figure 2:**
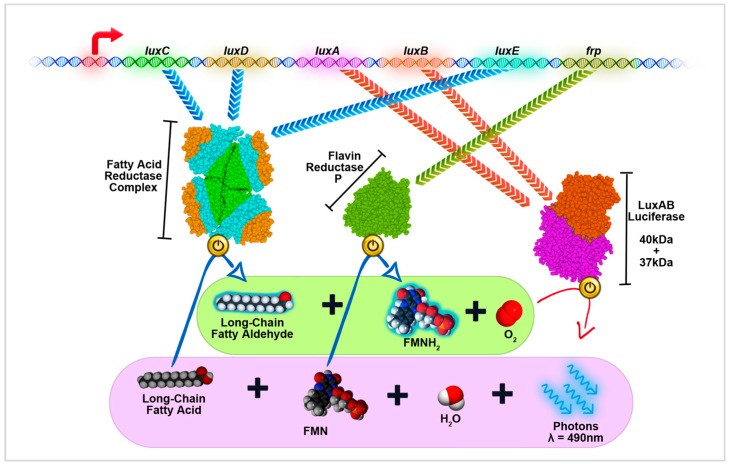
Schematic of a self-contained Lux operon system. The *lux* operon usually contains five different genes: *luxC*, *luxD*, *luxA*, *luxB*, and *luxE*. These five genes constitute the wildtype version of *lux* operon that can be found in *Photorhabdus luminescens*; this figure shows the modified version of *lux* operon that contains an additional *frp* gene for better operational efficiency when expressed in certain organisms. *luxA* and *luxB* code two subunits for dimeric LuxAB luciferase; *luxC*, *luxD*, and *luxE* code relative enzymes, which can form a dodecamer protein complex to produce fatty aldehyde substrate for LuxAB; and *frp* gene codes the Flavin reductase P that can be used to shift the natural cellular balance between FMN and FMNH_2_. In the presence of oxygen, LuxAB oxidizes fatty aldehyde and FMNH_2_ to generate cyan luminescence (λ = 490 nm). The oxidized products, fatty acid and FMN, can be recycled to re-form substrates. The continuous run of Lux system only requires the supplies of ATP, NADPH, and oxygen, so it is self-contained and able to constantly produce luminescence as long as the host cell is alive.

Within all of the known bioluminescent systems, bacterial luciferase system (Lux) is the only system that is self-contained and can produce both luciferase and its substrate with one operon [80]. Compared to other eukaryotic originated luciferase systems such as Rluc and Fluc, Lux system is completely different and evolved independently. The most typical Lux system is from *Photorhabdus luminescens*, and it is coded by *lux* operon, which consists of a series of five genes controlled by a single promoter (Figure 2). In *lux* operon, *luxA* and *luxB* code the bacterial luciferase LuxAB, which is a dimeric luciferase with two subunits sized at 40 and 37 kDa [81]. LuxAB catalyses the oxidization of long-chain fatty aldehyde, such as decanal, and FMNH_2_ to produce cyan coloured light that has a peak emission at 490 nm. The fatty aldehyde substrate can be synthesized by enzymes produced by the three other genes in *lux* operon, and FMNH_2_ widely exists in almost any organism; therefore, Lux is a very integrated system that can be ported to many species relatively easy. There are already many examples of expressing the whole Lux cassette in various organisms including some eukaryotes [82,83], but, on the other hand, as the actual bacterial luciferase LuxAB is only coded by two genes, it is also sensible to only port these two genes for better feasibility, and fatty aldehyde substrate can be added externally. In this case, due to the much simpler structure of fatty aldehyde compared to coelenterazine or d-luciferin, the substrate for LuxAB such as commonly used decanal, can be synthesized on an industrial scale and will cost almost nothing ($0.09 for 100 mg) [84]. Additionally, decanal has proven to be both membrane permeable and relatively non-toxic [85], so these features make LuxAB an attractive luciferase for use in BRET assays.

The use of endogenous or inexpensive substrate has caught researchers′ attention on Lux system. However, there are some significant drawbacks that limit the implementation of LuxAB in BRET. First, LuxAB is usually not as bright as Rluc or Fluc, though it has a relatively high quantum yield of 20% compared to 5.3% for native Rluc [86,87,88]. Second, it is a dimer and has a total size of 77 kDa, which is very unfavourable for protein fusion. Furthermore, Lux system had not been optimized for expression in mammalian cells until very recently [80]. Lastly, like many other luciferases, the blue emission of LuxAB is not good at tissue penetration.

Despite these hindrances, the substrate advantage of Lux system is unique and irreplaceable as the biosynthesis pathways for both coelenterazine and d-luciferin are unclear and numerous enzymes will be involved even if they are resolved someday [89,90]. In 2003, a mechanism study about bacterial luciferase shows that energy transfer between LuxAB and YFP is possible [91], and, in 2014, Cui et al. reported the first LuxAB based BRET system and it is proven to be useful and relatively reliable [28]. The system is designed only for bacteria, it uses the native *luxA* and *luxB* genes from *Photorhabdus luminescens*, and these two genes are cloned as a single combined part (not fused) into the same plasmid. One of the proteins of interest is fused to the C-terminus of *luxB*, and another is fused to the N-terminus of eYFP; both of these fusions use (GGGSG)_3_ linker. The substrate for the system is 1% decanal, which is manually added to the transformed culture before signal detection. This LuxAB-eYFP-decanal BRET system was validated in *E. coli* with several interaction models include rapamycin-induced dynamic interaction between FKBP12 and Frb. The FKBP12/Frb model is a simple but robust model that is often used to assess new PPI analytical methods [49], and, combined with two other interaction models, the authors demonstrated that the LuxAB based BRET system is fully capable of analysing various kinds of PPI interactions with rapid response, good sensitivity, and wide dynamic range. Although this system is currently only tested in *E. coli* and there are no further verifications or eukaryotic adaptations have been published yet (checked in July 2016), with many recent developments including creation of monomeric LuxAB luciferase [92] and efficient expression of whole Lux system in mammalian cell lines [80,83,93], the prevailing of LuxBRET is foreseeable.

## 5. Prospects of BRET

### 5.1. The Broader Selections of Fluorescent Proteins

The development of BRET assay is very dependent on the developments of its components, and FPs is the relatively mature part compared to luciferases. Since the original GFP was discovered more than a half century ago, different FPs have been used in numerous applications and undergone extensive research. Today, more than 1000 different kinds of FPs have been discovered or engineered with emissions that cover virtually every part of the visible spectrum from violet blue to infrared [94,95]. Among those, it is generally thought that the short-wave emitting FPs, including blue, green, and yellow FPs, are well exploited and not much further improvement can be made [96,97]. For example, many traditionally used FPs in BRET including eYFP, GFP^2^, mOrange, and tdTomato all have a quantum yield greater than 60% and are very bright; many recently developed FPs can even achieve more than 85% quantum yield, which is near perfect; in addition, most of them are stable, fast maturing, fusion compatible, and with consistent behaviour [94,95,96,97,98]. In contrast, red to infrared emitting FPs are less advanced with substantially lower brightness, or other problems; therefore, improving the performance of these FPs is the current focus of FP development [96,97]. With the remarkable advances toward this goal in recent years, such as the creations of FusionRed [99], smURFP [100], and iRFP [101], it is anticipated that red FPs that can perform as good as green or yellow FPs are on the horizon. At that time, highly capable red FPs can be adopted to further boost the deep layer performance of BRET.

On the other hand, there are also some explorations of not using FPs in BRET to avoid problems brought by gene fusion, and the corresponding protein of interest is labelled post-translationally. These studies include using quantum dots or specially engineered organic dyes to target small specific peptide sequences that are in or pre-fused to the proteins of interest [49,102,103]. However, they have some serious hindrances that limit their application, such as membrane impermeability, low RET efficiency, and off-targeting. These problems are unlikely to be solved anytime soon, so FPs will still be the most sensible acceptor choice for PPI BRET in the foreseeable future. With the growing number and variety of FPs, an online tool [95] can be helpful in choosing suitable FPs for different BRET assays.

### 5.2. Vast Development Potentials of Luciferases

Compared to FPs, luciferases have much room for improvement, and, as many luciferases have low quantum yield at around 10%, increasing brightness is an important part. Firefly luciferase was thought to have a very high quantum yield of 90% since the 1970s to early 2000s; however, recent research with more carefully designed measurements show that the actually quantum yield is only at about 41%, which is still the highest quantum yield of all known luciferases [104]. The quantum yield of super bright NanoLuc is not yet known, but it is supposed that the high brightness is due to the fast catalysing speed rather than the increase in quantum yield [70]. Therefore, it should be possible to get even brighter luciferases if their quantum yield can be increased to a reasonable level like FPs. Traditionally, the creation of new, improved luciferases are done by random mutagenesis, and rational design is nearly impossible due to the lack of understanding of its underlying mechanisms, which is also true for already highly developed FPs [98]. Though it will probably still be very difficult to do rational design for luciferases in the following decades, with the increasing computational power and experience from FP development, semi-rational mutagenesis may increase the efficiency in the exploration. Additionally, as the bright and small Gluc is largely unexploited, it can be an excellent start point for the progress.

Another important research direction is the low-cost or substrate free BRET with Lux system, which needs to be adapted into eukaryotes for wider application. The main obstacle of this is that eukaryotes have a different translation mechanism compared to bacteria: due to the encapsulated nucleus, an eukaryotic mRNA needs to be modified and transported to cytoplasm before it can be translated, and as a part of linked consequences, this lead to the abolishment of ribosomal binding site and the emergence of 5′ cap initiation, so only one kind of protein can be translated from a single mRNA [105]. Therefore, to directly transform the whole Lux system into eukaryotes, the construction will need at least five promoters and probably multiple plasmids, which is not only practically complicated but also greatly hinders the expression efficiency and consistency. In 2010, Close et al. tackled the problem by using bicistronic expression vectors and internal ribosomal entry site (IRES) element to simplify the construction [93], which still had some serious problems including low efficiency of transfection and expression. A real breakthrough came in 2014 by following the recent trend of utilizing viral 2A element in vector design [80]. 2A element is a small DNA sequence found in a virus that does not code a full protein but can be part of a protein coding sequence; when it is being expressed in eukaryotes, the corresponding peptide will disrupt the normal function of ribosome and cause one peptide bond in this region cannot be formed during translation, so the translated protein will fall apart at this location; additionally, it does not interfere with the ribosome’s other functions and the ribosome will continue to do its translation work along the mRNA chain as if nothing wrong was happened [106]. With 2A element, multiple polypeptides can be produced by one translation. In that 2014 research, all six genes (including *frp*) of the Lux system were fused together into a single open reading frame under the control of one promoter, and the corresponding stop codons are replaced with 2A linkers. The result of this approach is the efficient expressions of whole Lux system and autonomous bioluminescence in all tested human cell lines with just one plasmid construct [80].

This advancement opened the opportunity of creating substrate free LuxBRET systems in eukaryotes, which may be standardized in a variety of practical forms, such as with pre-designed Lux containing vectors or pre-engineered substrates producing cell lines. The 2A linker may also make the dimeric LuxAB more acceptable in BRET as it allowed the high-level co-expression of two subunits at a stable 1:1 ratio and more consistent complex forming. Nevertheless, the progress of creating monomeric LuxAB is not stopped, and a recent research shows that after mutagenesis and screening, linker fused monomeric LuxAB can achieve 60% brightness of dimeric LuxAB [92], which is actually quite usable in many situations.

### 5.3. Novel BRET Configurations and Applications

NanoBRET and LuxBRET might be the most popular BRET systems in several years, but they all have the problems brought by the short-wave emission of luciferase. For example, as the FPs′ excitation wavelength have a nearly linear correlation to their emission wavelength [95], most red FPs require yellow, or near red virtual photons to get excited, which cannot be directly provided by these luciferases. Solving this problem will require red-shift engineering of NanoLuc and LuxAB, or going back to Rluc8.6 or Fluc based systems. However, there might be another approach to evade the issue with Multistep or Tandem BRET (T-BRET) configuration. In T-BRET, constructed donor protein is a tri-fused protein with an additional FP part: it adopts a TargetProtein-FP-Luciferase fusion pattern where the additional FP is an intermediary coloured FP to relay the energy transfer from luciferase to acceptor red FP, and BRET ratio will be characterized by emissions from the two different FPs on donor and acceptor. Alternatively, the mediator FP could also be fused to acceptor protein in a similar way, or, if appropriate, with organic dyes or quantum dots added to the environment for energy relay. Though there is no published report about the exact same configurations yet, a similar cascaded energy transfer approach has already been successfully applied in FRET systems to address various issues [107,108,109,110,111]. Compared to conventional BRET, T-BRET approach could not only evade the acceptor excitation problem, but may also extend the interaction detection distance beyond 100 Å, or might be able to be used to study multi-branched large biomolecules if set up correctly [107].

Lastly, it is worth mentioning that, although this review is focused on BRET PPI assay, on the broader concept, however, BRET not only is used for PPI studies, but also has many other applications: a luciferase can be directly fused with one or multiple FPs or quantum dots to form long-wave self-luminescent particle for conventional biological imaging [111,112,113]; and a protein or short polypeptide fused with both luciferase and FP (or quantum dot) can be used to monitor or study protease activity [48,50,102], conformational changes [54,114,115], or used as real-time biological sensor [54,115]. With the developments of BRET systems, these applications could have improved performance and be easier to use in the future.

## 6. Recapitulation

The core of a BRET assay system is two distinct protein entities, one a luciferase tagged donor, and the other a fluorescent protein tagged acceptor. The donor can automatically produce light in the presence of a substrate, while acceptor usually remains dark. When these two proteins get very close to each other, the donor will contribute part of its energy to the acceptor and cause the acceptor to glow a different colour. In this way, the inter protein distance, which is invisible to the naked eye, will be converted to the measurable light signal for the study of protein–protein interactions. Compared to many other methods for analysing PPIs, BRET is highly sensitive and reliable, has relatively low cost and high-throughput, is widely applicable, and can be performed in living cells for real-time monitoring.

The principle of BRET assay is unchanged across different BRET systems and the development of the method in past seventeen years is mainly a progress of finding the best combinations of luciferase, FP, and substrate. The FP tag is the most mature part; it has a long application history across many different fields and the mechanism of FP is also much simpler than luciferase, so numerous FPs with all kinds of properties have been developed and can be selected fairly easily for different BRET assays in accordance with application. The options for luciferase and substrate are narrower: NanoLuc is best all-around luciferase for almost any application, but it may not be good for imaging in certain situations; Fluc or Rluc used to be good choices until the invention of NLuc, but they are still useful for imaging purposes with appropriate substrates; Gluc is a good alternative to Nluc if fully exploited; and LuxAB is a simple and inexpensive choice for BRET assay in bacteria, but it is not yet ready for use in eukaryotes.

The development of BRET assay depends on the advances of those general but essential elements, especially the luciferase. The creation of NanoLuc exhibits the power of intelligence guided evolution and indicates that the small protein size does not always mean less capability, so it is totally possible to create a simpler, smaller, and brighter Lux system for more versatile use in the following years. Additionally, the discovery of viral 2A elements, which is used in the eukaryotic adaptation of Lux system, shows that there are still many extremely valuable genetic resources that can be obtained from nature.

## Figures and Tables

**Table 1:** Comparisons between Bioluminescence Resonance Energy Transfer (BRET), Fluorescence Resonance Energy Transfer (FRET), Yeast Two-Hybrid (Y2H), Bimolecular Fluorescence Complementation (BiFC), and Co-Immunoprecipitation (Co-IP).

Method	Advantages	Disadvantages	Applications
BRET	Real-time PPI monitoring In vivo More sensitive than FRET	Requires expensive luciferin, but this might be solvable in the future with LuxBRET	PPI regulatory study Binary PPI detection
FRET	Real-time PPI monitoring In vivo	Requires external excitation Higher background noise Potential detriments to sample	PPI regulatory study Binary PPI detection
Y2H	Easy to use Low cost	Only suitable for proteins that are nucleus co-localizable Non-reversible detection In vivo only	Binary PPI detection
BiFC	Suitable for wider range of proteins than Y2H	Requires external excitation Non-reversible detection	Binary PPI detection
Co-IP	Detect multiple PPIs	Lower sensitivity Higher error rate In vitro only	Initial PPI screening Protein complex detection

**Table 2:** Summary of different BRET systems that can be used in PPI studies (Adapted and extended from Borroto-Escuela et al. [34]).

Name ^§^	Luciferase	Luciferase Emission *	FP	FP Emission *	Luciferin (Substrate)	Reference
BRET^1^	Rluc/Rluc8	480	eYFP	530	Coelenterazine-h	Xu et al. [39]
BRET^2^	Rluc	395	GFP^2^	510	DeepBlueC	Bertrand et al. [40]
eBRET^2^	Rluc8	395	GFP^2^	510	DeepBlueC	Kocan et al. [41]
BRET^3^ (1)	Rluc8	480	mOrange	564	Coelenterazine-h	De et al. [42]
BRET^3^ (2)	Fluc	565	DsRed	583	d-Luciferin	Gammon et al. [43]
eBRET	Rluc	480	eYFP	530	EnduRen	Pfleger et al. [44]
BAF-Y	Rluc	480	eYFP	530	ViviRen	Hoshino et al. [45]
BRET3.1	Rluc8	515	mOrange	564	Coelenterazine-v	Dragulescu et al. [46]
BRET4	Rluc8	480	TagRFP	584	Coelenterazine-h	Dimri et al. [47]
BRET4.1	Rluc8	515	TagRFP	584	Coelenterazine-v	Dragulescu et al. [46]
BRET5	Rluc8.6	535	TagRFP	584	Coelenterazine-h	Dragulescu et al. [46]
BRET6	Rluc8.6	535	TurboFP	635	Coelenterazine-h	Dragulescu et al. [46]
BRET6.1	Rluc8.6	570	TurboFP	635	Coelenterazine-v	Dragulescu et al. [46]
BRET7	Rluc8	515	TurboFP	635	Coelenterazine-v	Dimri et al. [47]
BRET8	Rluc8.6	535	TurboFP	635	Coelenterazine-h	Dimri et al. [47]
GlucBRET	hGluc	470	tdTomato	580	Coelenterazine	Li et al. [48]
NanoBRET	NLuc	460	HaloTag	618	Furimazine	Machleidt et al. [49]
LuxBRET	LuxAB	490	eYFP	530	FMNH_2_ + Long-chain fatty aldehyde	Cui et al. [28]
VlucBRET	Vluc	460	eYFP	530	Vargulin	Otsuji et al. [50]

^§^ These names are not absolute or exclusive, for reference only; * Peak wavelength in nm.

## References

[1] Vidal M., Cusick M.E., Barabasi A.L. (2011). Interactome networks and human disease. Cell.

[2] Chen K., Rajewsky N. (2007). The evolution of gene regulation by transcription factors and microRNAs. Nat. Rev. Genet..

[3] Hunke S., Müller V.S., Cai J. (2012). Approaches to analyze protein–protein interactions of membrane proteins. Protein Interactions.

[4] Licata L., Briganti L., Peluso D., Perfetto L., Iannuccelli M., Galeota E., Sacco F., Palma A., Nardozza A.P., Santonico E. (2012). MINT, the molecular interaction database: 2012 update. Nucleic Acids Res..

[5] Brückner A., Polge C., Lentze N., Auerbach D., Schlattner U. (2009). Yeast two-hybrid, a powerful tool for systems biology. Int. J. Mol. Sci..

[6] Berggård T., Linse S., James P. (2007). Methods for the detection and analysis of protein–protein interactions. Proteomics.

[7] LaBaer J., Ramachandran N. (2005). Protein microarrays as tools for functional proteomics. Curr. Opin. Chem. Biol..

[8] O’Connell M.R., Gamsjaeger R., Mackay J.P. (2009). The structural analysis of protein–protein interactions by NMR spectroscopy. Proteomics.

[9] Rao V.S., Srinivas K., Sujini G.N., Kumar G.N. (2014). Protein–protein interaction detection: Methods and analysis. Int. J. Proteom..

[10] Rowe L., Dikici E., Daunert S. (2009). Engineering bioluminescent proteins: Expanding their analytical potential. Anal. Chem..

[11] Subramanian C., Woo J., Cai X., Xu X., Servick S., Johnson C.H., Nebenführ A., von Arnim A.G. (2006). A suite of tools and application notes for in vivo protein interaction assays using bioluminescence resonance energy transfer (BRET). Plant J..

[12] Joly E., Houle B., Dionne P., Taylor S., Ménard L. Bioluminescence Resonance Energy Transfer (BRET^2^) Principles, Applications, and Products. http://web4.cbm.uam.es/joomla-rl/images/Servicios/070.Microscopia-optica-cfocal/documentos/manuales/bret.pdf.

[13] Helms V. (2008). Fluorescence resonance energy transfer. Principles of Computational Cell Biology.

[14] Scholes G.D. (2003). Long-range resonance energy transfer in molecular systems. Annu. Rev. Phys. Chem..

[15] Sapsford K.E., Berti L., Medintz I.L. (2006). Materials for fluorescence resonance energy transfer analysis: Beyond traditional donor-acceptor combinations. Angew. Chem. Int. Ed..

[16] Boute N., Jockers R., Issad T. (2002). The use of resonance energy transfer in high-throughput screening: BRET versus FRET. Trends Pharmacol. Sci..

[17] Morell M., Ventura S., Avilés F.X. (2009). Protein complementation assays: Approaches for the in vivo analysis of protein interactions. FEBS Lett..

[18] Kerppola T.K. (2008). Bimolecular fluorescence complementation (BiFC) analysis as a probe of protein interactions in living cells. Annu. Rev. Biophys..

[19] Luker G.D., Luker K.E. (2011). Luciferase protein complementation assays for bioluminescence imaging of cells and mice. Methods Mol. Biol..

[20] Li Y.C., Rodewald L.W., Hoppmann C., Wong E.T., Lebreton S., Safar P., Patek M., Wang L., Wertman K.F., Wahl G.M. (2014). A versatile platform to analyze low-affinity and transient protein–protein interactions in living cells in real time. Cell Rep..

[21] Wehr M.C., Laage R., Bolz U., Fischer T.M., Grünewald S., Scheek S., Bach A., Nave K.A., Rossner M.J. (2006). Monitoring regulated protein–protein interactions using split TEV. Nat. Methods.

[22] Ma Y., Nagamune T., Kawahara M. (2014). Split focal adhesion kinase for probing protein–protein interactions. Biochem. Eng. J..

[23] Dünkler A., Müller J., Johnsson N. (2012). Detecting protein-protein interactions with the split-ubiquitin sensor. Methods Mol. Biol..

[24] Remy I., Campbell-Valois F.X., Michnick S.W. (2007). Detection of protein–protein interactions using a simple survival protein-fragment complementation assay based on the enzyme dihydrofolate reductase. Nat. Protoc..

[25] Xie Q., Soutto M., Xu X., Zhang Y., Johnson C.H. (2011). Bioluminescence resonance energy transfer (BRET) imaging in plant seedlings and mammalian cells. Methods Mol. Biol..

[26] Pfleger K.D., Seeber R.M., Eidne K.A. (2006). Bioluminescence resonance energy transfer (BRET) for the real-time detection of protein-protein interactions. Nat. Protoc..

[27] Chen X., Zaro J.L., Shen W.C. (2013). Fusion protein linkers: Property, design and functionality. Adv. Drug Deliv. Rev..

[28] Cui B., Wang Y., Song Y., Wang T., Li C., Wei Y., Luo Z.Q., Shen X. (2014). Bioluminescence resonance energy transfer system for measuring dynamic protein–protein interactions in Bacteria. mBio.

[29] Marullo S., Bouvier M. (2007). Resonance energy transfer approaches in molecular pharmacology and beyond. Trends Pharmacol. Sci..

[30] Deriziotis P., Graham S.A., Estruch S.B., Fisher S.E. (2014). Investigating protein-protein interactions in live cells using bioluminescence resonance energy transfer. J. Vis. Exp..

[31] Mandić M., Drinovec L., Glisic S., Veljkovic N., Nøhr J., Vrecl M. (2014). Demonstration of a direct interaction between β2-adrenergic receptor and insulin receptor by BRET and bioinformatics. PLoS ONE.

[32] Szalai B., Hoffmann P., Prokop S., Erdélyi L., Várnai P., Hunyady L. (2014). Improved methodical approach for quantitative BRET analysis of G protein coupled receptor dimerization. PLoS ONE.

[33] Eidne K.A., Kroeger K.M., Hanyaloglu A.C. (2002). Applications of novel resonance energy transfer techniques to study dynamic hormone receptor interactions in living cells. Trends Endocrinol. Metab..

[34] Borroto-Escuela D.O., Flajolet M., Agnati L.F., Greengard P., Fuxe K. (2013). Bioluminescence resonance energy transfer methods to study G protein-coupled receptor-receptor tyrosine kinase heteroreceptor complexes. Methods Cell Biol..

[35] Promega Corporation NanoLuc^®^ Luciferase: Redefining Reporter Assays. https://www.promega.com/products/reporter-assays-and-transfection/reporter-assays/nanoluc-luciferase-redefining-reporter-assays.

[36] De A., Gambhir S.S. (2005). Noninvasive imaging of protein–protein interactions from live cells and living subjects using bioluminescence resonance energy transfer. FASEB J..

[37] Xu X., Soutto M., Xie Q., Servick S., Subramanian C., von Arnim A.G., Johnson C.H. (2007). Imaging protein interactions with bioluminescence resonance energy transfer (BRET) in plant and mammalian cells and tissues. Proc. Natl. Acad. Sci. USA.

[38] Goyet E., Bouquier N., Ollendorff V., Perroy J. (2016). Fast and high resolution single-cell BRET imaging. Sci. Rep..

[39] Xu Y., Piston D.W., Johnson C.H. (1999). A bioluminescence resonance energy transfer (BRET) system: Application to interacting circadian clock proteins. Proc. Natl. Acad. Sci. USA.

[40] Bertrand L., Parent S., Caron M., Legault M., Joly E., Angers S., Bouvier M., Brown M., Houle B., Ménard L. (2002). The BRET2/arrestin assay in stable recombinant cells: A platform to screen for compounds that interact with G protein-coupled receptors (GPCRS). J. Recept. Signal Transduct. Res..

[41] Kocan M., See H.B., Seeber R.M., Eidne K.A., Pfleger K.D. (2008). Demonstration of improvements to the bioluminescence resonance energy transfer (BRET) technology for the monitoring of G protein-coupled receptors in live cells. J. Biomol. Screen..

[42] De A., Ray P., Loening A.M., Gambhir S.S. (2009). BRET3: A red-shifted bioluminescence resonance energy transfer (BRET)-based integrated platform for imaging protein-protein interactions from single live cells and living animals. FASEB J..

[43] Gammon S.T., Villalobos V.M., Roshal M., Samrakandi M., Piwnica-Worms D. (2009). Rational design of novel red-shifted BRET pairs: Platforms for real-time single-chain protease biosensors. Biotechnol. Prog..

[44] Pfleger K.D., Dromey J.R., Dalrymple M.B., Lim E.M., Thomas W.G., Eidne K.A. (2006). Extended bioluminescence resonance energy transfer (eBRET) for monitoring prolonged protein-protein interactions in live cells. Cell Signal..

[45] Hoshino H., Nakajima Y., Ohmiya Y. (2007). Luciferase-YFP fusion tag with enhanced emission for single-cell luminescence imaging. Nat. Methods.

[46] Dragulescu-Andrasi A., Chan C.T., De A., Massoud T.F., Gambhir S.S. (2011). Bioluminescence resonance energy transfer (BRET) imaging of protein–protein interactions within deep tissues of living subjects. Proc. Natl. Acad. Sci. USA.

[47] Dimri S., Basu S., De A. (2016). Use of BRET to study protein-protein interactions in vitro and in vivo. Methods Mol. Biol..

[48] Li F., Yu J., Zhang Z., Cui Z., Wang D., Wei H., Zhang X.E. (2013). Use of hGluc/tdTomato pair for sensitive BRET sensing of protease with high solution media tolerance. Talanta.

[49] Machleidt T., Woodroofe C.C., Schwinn M.K., Méndez J., Robers M.B., Zimmerman K., Otto P., Daniels D.L., Kirkland T.A., Wood K.V. (2015). NanoBRET—A novel BRET platform for the analysis of protein–protein interactions. ACS Chem. Biol..

[50] Otsuji T., Okuda-Ashitaka E., Kojima S., Akiyama H., Ito S., Ohmiya Y. (2004). Monitoring for dynamic biological processing by intramolecular bioluminescence resonance energy transfer system using secreted luciferase. Anal. Chem..

[51] Wu N., Rathnayaka T., Kuroda Y. (2015). Bacterial expression and re-engineering of *Gaussia princeps* luciferase and its use as a reporter protein. Biochim. Biophys. Acta Proteins Proteom..

[52] Loening A.M., Wu A.M., Gambhir S.S. (2007). Red-shifted *Renilla reniformis* luciferase variants for imaging in living subjects. Nat. Methods.

[53] Trzaskowski B., Latek D., Yuan S., Ghoshdastider U., Debinski A., Filipek S. (2012). Action of molecular switches in GPCRs-theoretical and experimental studies. Curr. Med. Chem..

[54] Salahpour A., Espinoza S., Masri B., Lam V., Barak L., Gainetdinov R.R. (2012). BRET biosensors to study GPCR biology, pharmacology, and signal transduction. Front. Endocrinol..

[55] Kocan M., Pfleger K.D. (2011). Study of GPCR-protein interactions by BRET. Methods Mol. Biol..

[56] Stoddart L.A., Johnstone E.K., Wheal A.J., Goulding J., Robers M.B., Machleidt T., Wood K.V., Hill S.J., Pfleger K.D. (2015). Application of BRET to monitor ligand binding to GPCRs. Nat. Methods.

[57] Milligan G. (2004). Applications of bioluminescence-and fluorescence resonance energy transfer to drug discovery at G protein-coupled receptors. Eur. J. Pharm. Sci..

[58] Rebois R.V., Maki K., Meeks J.A., Fishman P.H., Hébert T.E., Northup J.K. (2012). D2-like dopamine and β-adrenergic receptors form a signaling complex that integrates G_s_-and G_i_-mediated regulation of adenylyl cyclase. Cell Signal..

[59] Biotium Inc. Physical Properties of Coelenterazine Analogs. http://www.bioscience.co.uk/userfiles/pdf/Physical-Properties-of-Coelenterazine-Analogs.pdf.

[60] Arai R., Nakagawa H., Kitayama A., Ueda H., Nagamune T. (2002). Detection of protein-protein interaction by bioluminescence resonance energy transfer from firefly luciferase to red fluorescent protein. J. Biosci. Bioeng..

[61] Thorne N., Inglese J., Auld D.S. (2010). Illuminating insights into firefly luciferase and other bioluminescent reporters used in chemical biology. Chem. Biol..

[62] De A., Jasani A., Arora R., Gambhir S.S. (2013). Evolution of BRET biosensors from live cell to tissue-scale in vivo imaging. Front. Endocrinol..

[63] Barnes A.T., Case J.F. (1972). Bioluminescence in the mesopelagic copepod, *Gaussia princeps* (T. Scott). J. Exp. Mar. Biol. Ecol..

[64] Verhaegen M., Christopoulos T.K. (2002). Recombinant *Gaussia* luciferase. Overexpression, purification, and analytical application of a bioluminescent reporter for DNA hybridization. Anal. Chem..

[65] Tannous B.A., Kim D.E., Fernandez J.L., Weissleder R., Breakefield X.O. (2005). Codon-optimized *Gaussia* luciferase cDNA for mammalian gene expression in culture and in vivo. Mol. Ther..

[66] Li F., Yu J., Zhang Z., Cui Z., Wang D., Wei H., Zhang X.E. (2012). Buffer enhanced bioluminescence resonance energy transfer sensor based on Gaussia luciferase for in vitro detection of protease. Anal. Chim. Acta.

[67] Kimura T., Hiraoka K., Kasahara N., Logg C.R. (2010). Optimization of enzyme-substrate pairing for bioluminescence imaging of gene transfer using *Renilla* and *Gaussia* luciferases. J. Gene Med..

[68] Inouye S., Shimomura O. (1997). The use of *Renilla* luciferase, *Oplophorus* luciferase, and apoaequorin as bioluminescent reporter protein in the presence of coelenterazine analogues as substrate. Biochem. Biophys. Res. Commun..

[69] Inouye S., Sasaki S. (2007). Overexpression, purification and characterization of the catalytic component of Oplophorus luciferase in the deep-sea shrimp, Oplophorus gracilirostris. Protein Expr. Purif..

[70] Hall M.P., Unch J., Binkowski B.F., Valley M.P., Butler B.L., Wood M.G., Otto P., Zimmerman K., Vidugiris G., Machleidt T. (2012). Engineered luciferase reporter from a deep sea shrimp utilizing a novel imidazopyrazinone substrate. ACS Chem. Biol..

[71] Riss T.L. NanoLuc^®^: A Smaller, Brighter, and More Versatile Luciferase Reporter. http://www.promega.com/~/media/files/promega%20worldwide/europe/promega%20uk/webinars%20and%20events/cell%20analysis%20seminar%20tour/terry-riss-02.pdf.

[72] England C.G., Ehlerding E.B., Cai W. (2016). NanoLuc: A small luciferase is brightening up the field of bioluminescence. Bioconjug. Chem..

[73] Los G.V., Encell L.P., McDougall M.G., Hartzell D.D., Karassina N., Zimprich C., Wood M.G., Learish R., Ohana R.F., Urh M. (2008). HaloTag: A novel protein labeling technology for cell imaging and protein analysis. ACS Chem. Biol..

[74] Promega Corporation Coelenterazines. https://www.promega.com/products/biochemicals-and-labware/biochemical-buffers-and-reagents/coelenterazines.

[75] Promega Corporation EnduRen™ Live Cell Substrate. http://www.promega.com/products/reporter-assays-and-transfection/reporter-assays/enduren-live-cell-substrate.

[76] Biotium Coelenterazine 400a (DeepBlueC). https://biotium.com/product/coelenterazine-400a-also-known-as-deepblue-ctm.

[77] Promega Corporation Nano-Glo^®^ Luciferase Assay System. http://www.promega.com/products/reporter-assays-and-transfection/reporter-assays/nano_glo-luciferase-assay-system.

[78] Sigma-Aldrich Co. d-Luciferin Synthetic. http://www.sigmaaldrich.com/catalog/product/sigma/l9504.

[79] Bhaumik S., Gambhir S.S. (2002). Optical imaging of *Renilla* luciferase reporter gene expression in living mice. Proc. Natl. Acad. Sci. USA.

[80] Xu T., Ripp S., Sayler G.S., Close D.M. (2014). Expression of a humanized viral 2A-mediated lux operon efficiently generates autonomous bioluminescence in human cells. PLoS ONE.

[81] Sharpe M.L., Hastings J., Krause K.L. (2014). Luciferases and light-emitting accessory proteins: Structural biology. eLS Encyclopedia of Life Sciences.

[82] Andreu N., Zelmer A., Fletcher T., Elkington P.T., Ward T.H., Ripoll J., Parish T., Bancroft G.J., Schaible U., Robertson B.D. (2010). Optimisation of bioluminescent reporters for use with mycobacteria. PLoS ONE.

[83] Close D., Xu T., Smartt A., Rogers A., Crossley R., Price S., Ripp S., Sayler G. (2012). The evolution of the bacterial luciferase gene cassette (*lux*) as a real-time bioreporter. Sensors.

[84] Sigma-Aldrich Co. Decanal. http://www.sigmaaldrich.com/catalog/product/sigma/d7384.

[85] Velten J., Pogson B., Cazzonelli C.I. (2008). Luciferase as a reporter of gene activity in plants. Transgenic Plant J..

[86] Close D.M., Xu T., Sayler G.S., Ripp S. (2010). In vivo bioluminescent imaging (BLI): Noninvasive visualization and interrogation of biological processes in living animals. Sensors.

[87] Welham P.A., Stekel D.J. (2009). Mathematical model of the Lux luminescence system in the terrestrial bacterium *Photorhabdus luminescens*. Mol. Biosyst..

[88] Loening A.M., Fenn T.D., Wu A.M., Gambhir S.S. (2006). Consensus guided mutagenesis of *Renilla* luciferase yields enhanced stability and light output. Protein Eng. Des. Sel..

[89] Oba Y., Kato S.I., Ojika M., Inouye S. (2009). Biosynthesis of coelenterazine in the deep-sea copepod, Metridia Pacifica. Biochem. Biophys. Res. Commun..

[90] Oba Y., Yoshida N., Kanie S., Ojika M., Inouye S. (2013). Biosynthesis of firefly luciferin in adult lantern: Decarboxylation of l-Cysteine is a key step for benzothiazole ring formation in firefly luciferin Synthesis. PLoS ONE.

[91] Low J.C., Tu S.C. (2003). Energy transfer evidence for in vitro and in vivo complexes of Vibrio harveyi flavin reductase P and luciferase. J. Photochem. Photobiol..

[92] Cui B., Zhang L., Song Y., Wei J., Li C., Wang T., Wang Y., Zhao T., Shen X. (2014). Engineering an enhanced, thermostable, monomeric bacterial luciferase gene as a reporter in plant protoplasts. PLoS ONE.

[93] Close D.M., Patterson S.S., Ripp S., Baek S.J., Sanseverino J., Sayler G.S. (2010). Autonomous bioluminescent expression of the bacterial luciferase gene cassette (*lux*) in a mammalian cell line. PLoS ONE.

[94] McNamara G., Boswell C.A., Méndez-Vilas A., Díaz J. (2007). A thousand proteins of light: 15 years of advances in fluorescent proteins. Modern Research and Educational Topics in Microscopy.

[95] Lambert T., Thorn K. Fluorescent Protein Properties. http://nic.ucsf.edu/FPvisualization.

[96] Kremers G.J., Gilbert S.G., Cranfill P.J., Davidson M.W., Piston D.W. (2011). Fluorescent proteins at a glance. J. Cell Sci..

[97] Chudakov D.M., Matz M.V., Lukyanov S., Lukyanov K.A. (2010). Fluorescent proteins and their applications in imaging living cells and tissues. Physiol. Rev..

[98] Dedecker P., De-Schryver F.C., Hofkens J. (2013). Fluorescent proteins: Shine on, you crazy diamond. J. Am. Chem. Soc..

[99] Shemiakina I.I., Ermakova G.V., Cranfill P.J., Baird M.A., Evans R.A., Souslova E.A., Staroverov D.B., Gorokhovatsky A.Y., Putintseva E.V., Gorodnicheva T.V. (2012). A monomeric red fluorescent protein with low cytotoxicity. Nat. Commun..

[100] Rodriguez E.A., Tran G.N., Gross L.A., Crisp J.L., Shu X., Lin J.Y., Tsien R.Y. (2016). A far-red fluorescent protein evolved from a cyanobacterial phycobiliprotein. Nat. Methods.

[101] Filonov G.S., Piatkevich K.D., Ting L.M., Zhang J., Kim K., Verkhusha V.V. (2011). Bright and stable near-infrared fluorescent protein for in vivo imaging. Nat. Biotechnol..

[102] Kim G.B., Kim Y.P. (2012). Analysis of protease activity using quantum dots and resonance energy transfer. Theranostics.

[103] Yano Y., Matsuzaki K. (2009). Tag–probe labeling methods for live-cell imaging of membrane proteins. Biochim. Biophys. Acta Biomembr..

[104] Ando Y., Niwa K., Yamada N., Enomoto T., Irie T., Kubota H., Ohmiya Y., Akiyama H. (2008). Firefly bioluminescence quantum yield and colour change by pH-sensitive green emission. Nat. Photonics.

[105] Jackson R.J., Hellen C.U., Pestova T.V. (2010). The mechanism of eukaryotic translation initiation and principles of its regulation. Nat. Rev. Mol. Cell Biol..

[106] Kim J.H., Lee S.R., Li L.H., Park H.J., Park J.H., Lee K.Y., Kim M.K., Shin B.A., Choi S.Y. (2011). High cleavage efficiency of α 2A peptide derived from porcine teschovirus-1 in human cell lines, zebrafish and mice. PLoS ONE.

[107] Saha J., Dey D., Roy A.D., Bhattacharjee D., Hussain S.A. (2016). Multi step FRET among three laser dyes Pyrene, Acriflavine and Rhodamine B. J. Lumin..

[108] Aneja A., Mathur N., Bhatnagar P.K., Mathur P.C. (2008). Triple-FRET technique for energy transfer between conjugated polymer and TAMRA dye with possible applications in medical diagnostics. J. Biol. Phys..

[109] Alam R., Zylstra J., Fontaine D.M., Branchini B.R., Maye M.M. (2013). Novel multistep BRET-FRET energy transfer using nanoconjugates of firefly proteins, quantum dots, and red fluorescent proteins. Nanoscale.

[110] Jiang G., Susha A.S., Lutich A.A., Stefani F.D., Feldmann J., Rogach A.L. (2009). Cascaded FRET in conjugated polymer/quantum dot/dye-labeled DNA complexes for DNA hybridization detection. ACS Nano.

[111] Xiong L., Shuhendler A.J., Rao J. (2012). Self-luminescing BRET-FRET near-infrared dots for in vivo lymph-node mapping and tumour imaging. Nat. Commun..

[112] So M.K., Xu C., Loening A.M., Gambhir S.S., Rao J. (2006). Self-illuminating quantum dot conjugates for in vivo imaging. Nat. Biotechnol..

[113] Kosaka N., Mitsunaga M., Bhattacharyya S., Miller S.C., Choyke P.L., Kobayashi H. (2011). Self-illuminating in vivo lymphatic imaging using a bioluminescence resonance energy transfer quantum dot nano-particle. Contrast Media Mol. Imaging.

[114] Wang J., Ren J., Wu B., Feng S., Cai G., Tuluc F., Peränen J., Guo W. (2015). Activation of Rab8 guanine nucleotide exchange factor Rabin8 by ERK1/2 in response to EGF signaling. Proc. Natl. Acad. Sci. USA.

[115] Sleno R., Pétrin D., Devost D., Goupil E., Zhang A., Hébert T.E. (2016). Designing BRET-based conformational biosensors for G protein-coupled receptors. Methods.

